# Dietary Bile Acid Influences the Physiological, Morphological, Lipid Metabolism-Related Responses, and Transcriptomic Profile of Hepatopancreas in High-Fat Diet-Fed Juvenile Gibel Carp (*Carassius auratus gibelio*)

**DOI:** 10.3390/ani15192853

**Published:** 2025-09-30

**Authors:** Xiaoyang Huang, Zikui Yang, Xiangning Chen, Jingjing Zhang, Yanru Wu, Huiqing Li, Haiming Yuan, Rui Feng, Chaoqing Wei, Zhujin Ding, Jianhe Xu, Hanliang Cheng

**Affiliations:** 1 Jiangsu Key Laboratory of Marine Bioresources and Environment/Jiangsu Key Laboratory of Marine Biotechnology, Jiangsu Ocean University, Lianyungang 222005, China; 2023210123@jou.edu.cn (X.H.);; 2 Co-Innovation Center of Jiangsu Marine Bio-Industry Technology, Jiangsu Ocean University, Lianyungang 222005, China; 3 College of Marine Science and Fisheries, Jiangsu Ocean University, Lianyungang 222005, China; 4 Wuxi Fisheries College, Nanjing Agricultural University, Wuxi 214081, China

**Keywords:** gibel carp, bile acids, high-fat diet, hepatopancreas, lipid homeostasis, RNA-seq

## Abstract

To assess the influence of dietary bile acid (BA) on the phenotype associated with hepatic lipid metabolism and its regulation of lipid homeostasis in gibel carp (*Carassius auratus gibelio*) under high-fat diet (HFD) conditions, five HFDs were designed using soybean oil (SO) as the single lipid source and supplemented with 0, 200, 400, 600, and 800 mg/kg BA (designated as BA0, BA200, BA400, BA600, and BA800, respectively). Juvenile fish (32.37 ± 0.13 g) were fed five BA-added HFDs (12% SO) for 8 weeks. Considerably lower levels of aspartate transaminase, alanine aminotransferase, low-density lipoprotein, triglyceride, and total cholesterol in the serum were observed in gibel carp fed with HFDs with 400–600 mg/kg BA (*p* < 0.05). The hepatocytes of the BA400 and BA600 groups were intact without abnormal architecture or histopathological changes, compared to other groups. The presence of most genes related to fatty acid biosynthesis decreased significantly with the addition of 400–600 mg/kg BA (*p* < 0.05), while the gene expressions of hormone-sensitive lipase, adiponectin receptor 2, and peroxisome proliferator-activated receptor *α* were variably up-regulated, along with the elevation of dietary BA (*p* < 0.05). Critical genes involved in bile acid and cholesterol synthesis were obviously down-regulated in gibel carp receiving 600–800 mg/kg dietary BA (*p* < 0.05), despite the sterol 27-hydroxylase (*cyp27a1*) gene in the BA800 group (*p* < 0.05). Moreover, hepatopancreas from the BA0 and BA600 groups were isolated for transcriptome sequencing, identifying 7040 differentially expressed genes (DEGs). The enriched KEGG pathways of DEGs mainly included steroid biosynthesis, protein digestion and absorption, etc. Seven randomly selected DEGs were validated using qRT-PCR and were in agreement with the RNA-seq results. Consequently, the appropriate supplementation of dietary BA for juvenile gibel carp is recommended at doses of 400–600 mg/kg in SO-based HFDs, which could contribute to the amelioration of HFD-induced excessive fat deposition in the hepatopancreas of gibel carp by both inhibiting fatty acid intake, biosynthesis, and steroid production and enhancing lipid decomposition. The findings may elucidate the physiological role of exogenous BA in fish and its underlying mechanism, providing references for the reasonable application of BA in aquafeeds and the prevention of HFD-induced metabolic dysfunction in fish.

## Simple Summary

Despite many advantages in aquaculture presented by a high-fat diet (HFD), long-term HFD feeding can impair growth and health in fish. Bile acids (BAs), as feed additives, have been reported to alleviate the HFD-induced metabolic disorders in fish. But the specific effects of BA vary with aquatic animals, and its underlying mechanism should be further elucidated. Therefore, this study focused on the impact of BA on the physiological and transcriptional changes associated with hepatic lipid metabolism, as well as transcriptomic characteristics in HFD-fed gibel carp (*Carassius auratus gibelio*). Fish fed on diets with 400–600 mg/kg BA exhibited obviously lower contents of aspartate transaminase, alanine aminotransferase, low-density lipoprotein, triglyceride, and total cholesterol, which was accompanied by the relief of liver pathological injury. In HFD-fed gibel carp, BA addition of 400–600 mg/kg (particularly 600 mg/kg) not only significantly down-regulated genes in lipogenesis and cholesterol synthesis but also significantly increased genes in lipid catabolism and transport. Using RNA-seq, 7040 differentially expressed genes were identified between fish receiving 0 and 600 mg/kg dietary BA and were mainly enriched in the steroid metabolism pathway. These findings may provide valuable information regarding the application of BA in aquafeeds and the molecular mechanism of BA relieving metabolic dysfunction in HFD-fed fish.

## 1. Introduction

Driven by the continuous expansion of global aquaculture, the ever-increasing need for protein sources, the tight supply of fish meal, and the high reliance on imported feed protein can further exacerbate the increasing costs and the developmental challenges in aquaculture [1,2], particularly in China. Thus, more available and cheaper alternative feeds are necessarily explored and used in aquafeed to fulfill their energy requirements. Among multiple alternative raw materials to protein, high lipid-based ingredients have received high attention and have been widely adopted in aquaculture due to the higher energy density, low cost, and essential role in various physiological activities in fish [3,4]. Despite the multiple advantages in aquaculture presented by this elevation in dietary fat, such as cost-effectiveness and low nitrogen excretion [4,5], the continuous overconsumption of dietary fat or misuse of a high-fat diet (HFD) would increase the risk of hepatic steatosis, fatty liver, and other metabolic dysfunction in fish, thereby compromising the growth, health, and quality of aquatic products [6,7,8,9,10]. Given the extensive application of HFDs in aquaculture, developing a feed additive to mitigate lipid metabolism disorder and the related adverse effects in aquatic animals, particularly in the core metabolic organ, the liver, is a priority.

Bile acids (BAs) are a primary component of bile and a class of amphosterols synthesized from cholesterol in the liver [10]. They can facilitate the emulsification of lipids and the formation of mixed micelles, which in turn contribute to the digestion and absorption of lipids, cholesterol, and fat-soluble vitamins [11]. BAs have been verified as effective feed additives with benefits in growth, nutrient metabolism, and immune function in multiple aquatic animals, especially those fed high-energy diets [10,12,13,14]. BAs can also function as signaling molecules through PPARs and relevant nuclear hormone receptor-mediated pathways [14,15]. The recommended dose of BAs supplemented in fish feed ranges from 250 to 1500 mg/kg [16]. Nevertheless, it is worth highlighting that the detailed effects of exogenous BAs vary among different species of aquatic animals, tissues of the same species, and BA types [10,15,16,17]. For example, the supplementation of dietary BA at 60–600 mg/kg showed a positive effect on common carp (*Cyprinus carpio* L.) [17], whereas the promoting effect of BA was found in largemouth bass (*Micropterus salmoides*) fed 900 mg/kg BA supplemented in HFDs [10]. Therefore, the complex mechanisms underlying the BA-supplied effects need to be further clarified.

Gibel carp (*Carassius auratus gibelio*) is a typical omnivorous species with high populations in Eurasian countries [18]. It is one of the major economic freshwater fish in China due to its fast growth, good environmental adaptability, high yield, and rich nutrition [18,19]. The adverse effects induced by HFD feeding have been evidenced in many fish [6,7,8,20], including gibel carp and other cyprinid fishes, particularly under intensive aquaculture conditions. Functional/nutritional additives in aquafeeds have been demonstrated to be a viable approach to palliating HFD-induced metabolic and growth disturbances, thereby improving various aspects of fish farming [9,12,20,21]. A study has reported that the suitable concentration and the tolerated concentration of dietary BA were 60 mg/kg and 600 mg/kg for common carp fed on diets with low dietary lipid content, respectively [17]. In our previous work, the addition of 400–600 mg/kg BA to HFDs promoted the growth performance in gibel carp [22], accompanied by a significant increase in crude protein content in muscle. Nevertheless, there are limited studies on the incorporation of BAs into gibel carp feed and their overall effects on the physiological, morphological, and molecular changes associated with lipid metabolism. Moreover, the genetically improved breed, gibel carp “CAS III”, is among the gibel carp varieties with good attributes and is used in the majority of gibel carp production in China [23]. Given that the lipid-lowering effect of BA is highly related to dietary lipid contents [16], the reported optimal level of BA in some Cyprinidae fish species [17,24], as well as the high lipid levels employed in the diets of this study (about 12% dietary lipid level), this study aimed to investigate the influence of dietary BAs with five different contents on biochemical responses, hepatopancreas histology, lipid metabolism-related gene expression, and transcriptomic characteristics in HFD-fed gibel carp using CAS III. Our results would benefit the further utilization of exogenous BAs as feed additives in gibel carp, which may also offer guidance for developing low-cost, efficient, and environmentally friendly compound feed for gibel carp, alongside ameliorating lipid metabolism-associated issues in HFD-fed gibel carp and other fish species.

## 2. Materials and Methods

### 2.1. Ethics Statement

All experimental operations met the animal ethical principles and welfare for experimental animals in China and were authorized by the Animal Care and Utilization Committee of Jiangsu Ocean University (No. 2020-37).

### 2.2. Experimental Feed

Five isonitrogenous (35% crude protein) and isolipidic (12% crude fat) diets were prepared to contain graded levels of bile acid (BA) (Shandong Longchang Animal Health Products Co., Ltd., Jinan, China). Soybean oil was used as the single lipid source, while fish meal, soybean meal, cottonseed meal, and canola meal were used as the protein sources. BA powder was used for the supplements at a concentration of 0, 200, 400, 600, and 800 mg/kg (named as BA0, BA200, BA400, BA600, and BA800, respectively). The formulation and nutritional composition of the experimental diets are presented in Table 1.

After the preparation was complete, all the experimental feedstuffs were dried at 55–60 °C, packed, sterilely sealed in plastic containers, and stored at 4 °C for the subsequent feeding trial. The detailed procedures of feed preparation were performed following our previous study [4].

### 2.3. Fish Management and Feeding Experiment

All juvenile gibel carp (*Carassius auratus gibelio* var. CAS III) were acquired from the Huandun fishery (Lianyungang, Jiangsu Province, China) and quickly transported to the Jiangsu Key Laboratory of Marine Biotechnology, Jiangsu Ocean University (Lianyungang, Jiangsu Province, China). Before the feeding experiment, gibel carp were temporarily reared in indoor flow-through tanks (240 L/tank) to adapt to the laboratory environment for 2 weeks. During acclimatization, fish were fed on the control diet (BA0) 4 times a day (8:00, 11:00, 14:00, and 17:00) to apparent satiation. Feed residues and feces were removed by siphoning after 45–60 min of feeding. Water quality was monitored regularly and maintained within the following range: water temperature 20–25 °C, dissolved oxygen greater than 5.5 mg/L, pH value 7–8, ammonia nitrogen less than 0.12 mg/L, and photoperiod 12 h light–12 h dark cycle.

After the 14-day acclimation, 450 healthy and uniform-sized gibel carp (initial body weight: 32.37 ± 0.13 g, age: 2 months) were randomly allocated to 5 groups with three 240 L replicate tanks (30 individuals/replicate tank). Fish were artificially fed with the respective BA-supplemented diets (4 times/day) until full satiation for 8 weeks. The uneaten food and feces were removed by siphoning when observing feed remained after 45–60 min of feeding. Throughout the feeding trial, the rearing and aquatic conditions were consistent with the acclimation period.

### 2.4. Sample Collection

At the end of the 8-week feeding experiment, gibel carp were fasted for 24 h to minimize postprandial and food interference in serum biochemical analyses, facilitate the emptying of the digestive tract, and remove any possible contamination of tissue samples during sampling. The fasted individuals were anesthetized with 20 mg/L MS-222 (Merck KgaA, Darmstadt, Germany). Six fish were randomly selected in each group (two fish/tank, three tanks/group) and weighed individually. Blood was sampled from the caudal vein of these sedated fish, centrifuged at 3000 rpm at 4 °C for 15 min using a refrigerated centrifuge (FTC-29R, Thermo Fisher Scientific, Wilmington, DE, USA), and the supernatant was kept at −80 °C for the serum biochemical indexes. After this quick dissection, the hepatopancreas of the selected fish were separated, flash-frozen with liquid nitrogen, and placed at −80 °C for gene expression evaluation and transcriptome sequencing. Another three hepatopancreatic samples from each tank were obtained and fixed in a centrifuge tube containing 4% paraformaldehyde (Beijing Solarbio Science & Technology Co., Ltd., Beijing, China) at 4 °C for further histological analysis.

### 2.5. Serum Biochemical Indexes

The serum concentrations of high-density lipoproteins, low-density lipoproteins, triglycerides, and total cholesterol and the activities of aspartate transaminase and alanine aminotransferase in serum were assessed using the corresponding diagnostic kits (Nanjing Jiancheng Bioengineering Institute, Nanjing, China). The specific operation procedure of each plasma biochemical parameter was completed on a microplate reader (Thermo Scientific, Waltham, MA, USA), strictly following the manufacturer’s manual.

### 2.6. Slice Preparation and Histological Observation

The hepatopancreatic paraffin sections and staining procedure were conducted based on the previously described method [25]. The main steps are outlined as below: (1) dehydrate in the gradient ethanol (75–100%); (2) clear with xylene twice; (3) embed in paraffin; (4) trim and cut to 5 μm thickness using a Leica tissue slicer; (5) dry at 60 °C; (6) deparaffinize in xylene and the gradient ethanol (100–75%); (7) wash with distilled water; (8) dye with hematoxylin solution for 5 min; (9) wash with tap water and incubate in a saturated lithium carbonate solution for 10–15 s; (10) dye with 0.5% eosin solution for 1 min; (11) dehydrate with anhydrous ethanol twice; (12) clear with xylene twice; and (13) mount with neutral gum. The stained sections were visualized and photographed with a NIKON Microscope (ECLIPSE Ci-L, Nikon, Tokyo, Japan).

### 2.7. RNA Extraction, Library Sequencing, and Transcriptome Assembly

Based on the overall biochemical, morphological, and transcriptional alterations, the BA-induced mitigation effect on liver damage in HFD-fed gibel carp is more marked in fish receiving 600 mg/kg dietary BA than in those in other groups. Three hepatopancreatic tissues of gibel carp in the BA0 and BA600 groups were obtained and mixed as one specimen for transcriptomic analysis, respectively. The total RNA of six hepatopancreas mixed samples (two dietary BA levels × three biological replicates) was extracted following the manufacturer’s instructions. The isolated RNA was qualified and quantified using 1.5% agarose gel electrophoresis and an Agilent 2100 Bioanalyzer (Agilent Technologies, Santa Clara, CA, USA), respectively.

The extracted RNA samples with high purity and high RIN scores (>7.8) were used for the library construction and sequencing, which was performed by Sangon Biotech Co., Ltd. (Shanghai, China) on the Illumina HiSeq-2500 platform.

Raw sequencing data were evaluated using the FastQC software (version 0.11.2) and then trimmed with the Trimmomatic software (version 0.36) to remove readers with adaptor-polluted, low-quality, and unknown bases. The valid and clean reads were mapped to the reference genome using HISAT2 2.1.0 (http://www.ccb.jhu.edu/software/hisat (accessed on 25 September 2023)) and then further analyzed using the RSeQC package (version 2.6.1). The successfully aligned reads were assembled by StringTie (v2.2.3), followed by a comparison with known transcripts and the identification of novel transcripts using GffCompare (v0.10.1). Raw data of transcriptome sequencing were uploaded and deposited in the NCBI sequence read archive (SRA) database (Accession No. PRJNA1261596).

### 2.8. Identification and Functional Annotation of Differentially Expressed Genes (DEGs)

The expression abundances of the mapped reads were represented as TPM (Transcripts Per Million) values and quantified based on the known gene model. DEGs were identified using the DEGseq R package (version 1.26.0) for hepatopancreatic samples between the BA0 and BA600 groups (three samples in each group). The parameters for significant differential expression were adjusted as follows: *p* < 0.05 and |log2 (Fold Change)| > 1. The topGO R package (version 2.24.0) was applied for Gene Ontology (GO) enrichment analysis. Meanwhile, the Kyoto Encyclopedia of Genes and Genomes (KEGG) pathway enrichment analysis of DEGs was accomplished using clusterProfiler (version 3.0.5). GO terms and KEGG pathways with *p* < 0.05 were considered to be significantly enriched.

### 2.9. Quantitative Real-Time Polymerase Chain Reaction (qRT-PCR) Analysis

Isolated total RNA in the hepatopancreatic sample was reverse-transcribed to the first-strand cDNA using FastKing gDNA Dispelling RT SuperMix (KR118-02, Tiangen Biotech Co., Ltd., Beijing, China). The qRT-PCR reactions of lipid metabolism-related genes and representative DEGs were conducted using TOROGreen^®^ qPCR Master Mix (QST-100, Toroivd, Toroivd Technology Company Limited, Shanghai, China) on a StepOne Plus Real-Time PCR system (Applied Biosystems, Foster City, CA, USA). The reaction system and amplification procedure of qRT-PCR were set according to the method of our previous research [26], with modifications.

Briefly, the 20 μL reaction mixture contained 10 μL TOROGreen^®^ qPCR Master Mix, 0.4 µL forward primer (25 µmol/L), 0.4 µL reverse primer (25 µmol/L), 2 µL cDNA template, and 7.2 µL RNase-free water. The cycling parameters were set as follows: pre-denaturation at 95 °C for 1 min, 40 cycles of 95 °C, 58 °C, and 72 °C for 15 s, 15 s, and 45 s, respectively, and extension at 95 °C, 60 °C, and 72 °C for 15 s, 1 min, and 15 s, respectively. Each qRT-PCR system contained a negative control without a cDNA template and was conducted in three replicates for the respective sample (hepatopancreatic tissue from three fish per tank mixed as a composite sample). qRT-PCR primers were synthesized by Sangon (Shanghai, China), and the corresponding primer information is presented in Table 2. Elongation factor 1 alpha (*ef1a*) and ribosomal protein L13a (*rpl13a*) genes were set as the internal reference genes because of their steady expressions in this study. The relative abundance of each target gene was computed via the 2^−ΔΔCt^ method.

### 2.10. Statistical Analysis of Experimental Data

Data were pooled and presented as mean ± standard deviation (SD) if the data met normal distribution. The comparative analyses among groups were conducted by one-way analysis of variance (ANOVA) coupled with Tukey’s or LSD post hoc test. Statistical processing of all data was carried out using SPSS 23.0 software (SPSS Inc., Chicago, IL, USA). The figures of all data were drawn using GraphPad Prism 8 software (GraphPad, La Jolla, CA, USA). Statistical significance was set to *p* < 0.05.

## 3. Results

### 3.1. Serum Biochemical Indices in Gibel Carp Fed with Various Levels of Dietary BA

**Figure 1:**
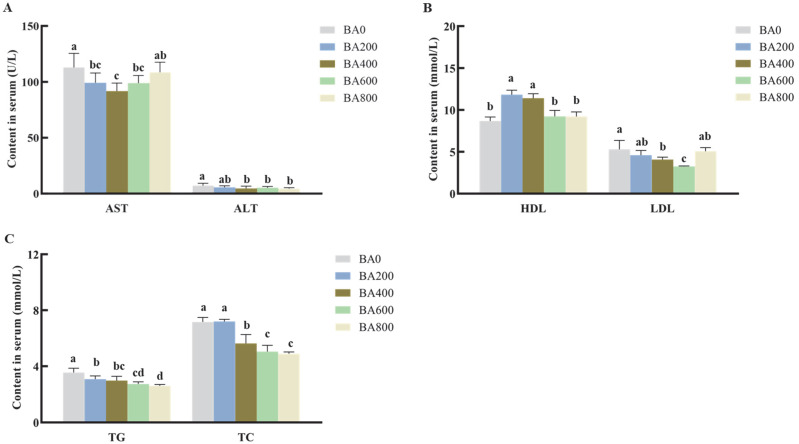
Serum biochemical parameters of gibel carp fed with high-fat diets (HFDs) containing various levels of bile acid (BA) for 8 weeks. Average content of AST and ALT (**A**), HDL and LDL (**B**), and TG and TC (**C**) in the serum of gibel carp fed on HFDs with various levels of BA. All values are shown as mean ± SD (*n* = 4–6) and further evaluated via one-way ANOVA. Bars with different letters represent a significant difference between groups (*p* < 0.05). AST: aspartate aminotransferase; ALT: alanine aminotransferase; HDL: high-density lipoprotein cholesterol; LDL: low-density lipoprotein cholesterol; TG: triglycerides; TC: total cholesterol.

As shown in Figure 1, serum ALT progressively decreased to the lowest activity with the increasing dietary BA (*p* < 0.05). Similarly, a significant decline in serum AST and LDL was found in gibel carp receiving a 200–600 mg/kg BA-supplemented diet compared with that in the BA0 group (*p* < 0.05). But both the AST and LDL content in the serum increased in fish from the BA800 group and recovered to the level of the BA0 group (*p* < 0.05). The obviously higher concentration of serum HDL was observed in the BA200 and BA400 groups (*p* < 0.05; Figure 1). Serum TG and TC levels were substantially decreased when dietary BA supplementation was elevated from 200 mg/kg to 800 mg/kg (*p* < 0.05), except serum TC in the BA200 group (*p* > 0.05).

### 3.2. Histopathological Observation of the Hepatopancreas in Gibel Carp Fed with Various Levels of Dietary BA

**Figure 2:**
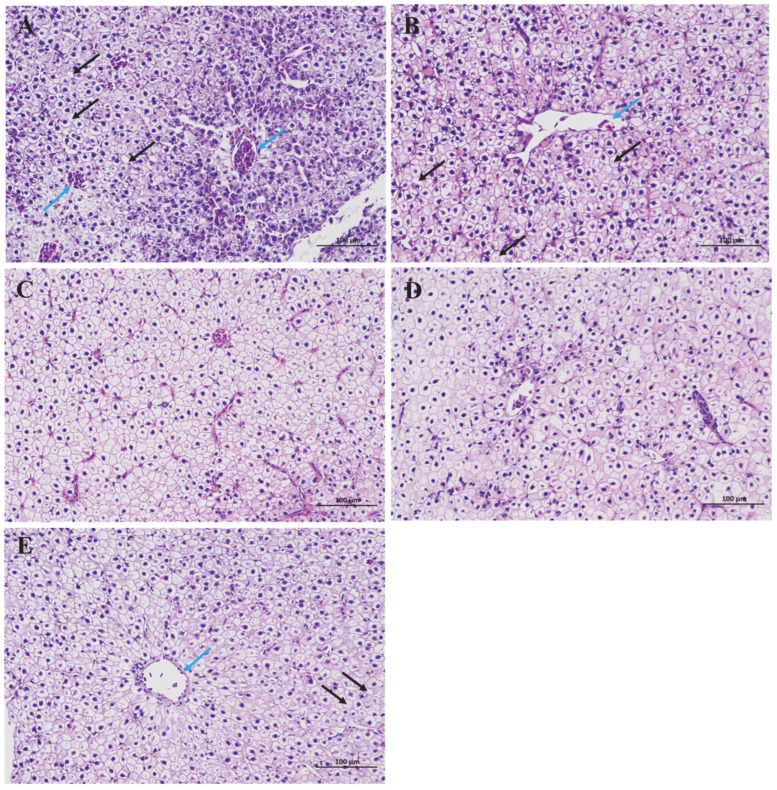
Effects of various BA levels on the hepatopancreatic morphology of HFD-fed gibel carp. Representative histological image of the hepatopancreas of gibel carp fed on HFDs with 0 mg/kg (**A**), 200 mg/kg (**B**), 400 mg/kg (**C**), 600 mg/kg (**D**), and 800 mg/kg (**E**) BA. The histopathological sections were stained with hematoxylin and eosin (HE) solution. The black arrow represents the cytoplasmic vacuolation. The blue arrow represents the location of lymphocytic infiltration. Scale bar: 100 µm.

Hepatocytes in the BA0 group were densely arranged, exhibiting occasional fat vacuolization (black arrow) in local hepatopancreatic tissue (Figure 2). But the marked congestion and lymphocyte infiltration (blue arrow) were clearly visible in the central vein, hepatic sinusoid, and the perisinusoidal spaces of the BA0 group (Figure 2). The BA200 group showed a significantly lower degree of hyperemia and inflammation in the hepatopancreas (Figure 2). The congestion and inflammatory phenomena further decreased in hepatopancreas with the increase in the dietary BA content to 400–600 mg/kg (Figure 2). However, more vacuolation and fewer nuclei were observed in the hepatopancreas of the BA400 group (Figure 2). Nucleus migration and a small amount of congestion were observed in the central vein of the BA800 group, accompanied by smaller hepatocytes and evident vacuolar degeneration (Figure 2).

### 3.3. Relative Expression of Lipid Metabolism-Related Genes in the Hepatopancreas of Gibel Carp Fed with Various Levels of Dietary BA

**Figure 3:**
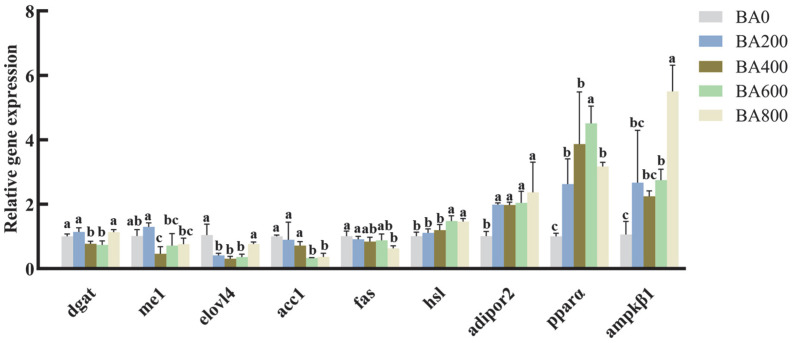
Effects of various BA levels on the expressions of lipid metabolism-related genes in the hepatopancreas of HFD-fed gibel carp. Transcriptional expressions of genes for lipid metabolism in the hepatopancreas of gibel carp fed on HFDs with various levels of BA. The relative expression of each gene was measured by quantitative real-time PCR (qRT-PCR) and calculated for the expression of the candidate housekeeping genes (*ef1a* and *rpl13a*). All values are shown as mean ± SD (*n* = 6) and further evaluated via one-way ANOVA. Bars with different letters represent a significant difference between groups (*p* < 0.05).

Relative mRNA levels of diacylglycerol acyltransferase (*dgat*), malic enzyme 1 (*me1*), and elongase of very long-chain fatty acids 4 (*elovl4*) genes were significantly lower at 400–600 mg/kg of BA addition (*p* < 0.05), while their expressions increased variously in the BA800 group and returned to the levels nearly equivalent to the control group (*p* < 0.05). Acetyl-CoA carboxylase 1 (*acc1*) and fatty acid synthase (*fas*) genes were gradually down-regulated with increasing dietary BA content, showing the lowest abundance in the BA600 and BA800 group, respectively (*p* < 0.05; Figure 3). As the BA addition contents increased, the transcription levels of lipoprotein lipase (*hsl*), adiponectin receptor 2 (*adipor2*), and AMP-activated protein kinase β1 (*ampkβ1*) increased incrementally to their respective maximum (*p* < 0.05; Figure 3). The gene abundance of peroxisome proliferator-activated receptor α (*ppar α*) considerably enhanced along with dietary BA usage from 0 mg/kg to 800 mg/kg (*p* < 0.05; Figure 3), peaking in the BA600 group.

**Figure 4:**
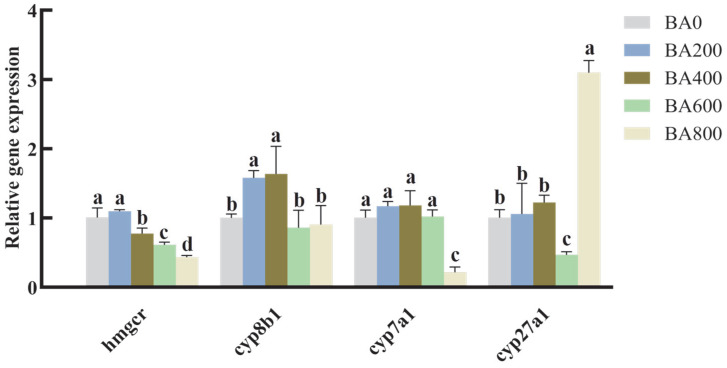
Effects of various BA levels on the expressions of BA metabolism-related genes in the hepatopancreas of HFD-fed gibel carp. Transcriptional expressions of genes for BA and cholesterol metabolism in the hepatopancreas of gibel carp fed on HFDs with various levels of BA. The relative expression of each gene was measured by quantitative real-time PCR (qRT-PCR) and calculated for the expression of the candidate housekeeping genes (*ef1a* and *rpl13a*). All values are shown as mean ± SD (*n* = 6) and further evaluated via one-way ANOVA. Bars with different letters represent a significant difference between groups (*p* < 0.05).

Regarding the key genes involved in BA synthesis and cholesterol metabolism, the addition of dietary BA in the range of 400 to 800 mg/kg caused a significantly reduced 3-hydroxy-3-methylglutaryl-CoA reductase (*hmgcr*) mRNA level. The down-regulation of cytochrome P450 7A1 (*cyp7a1*) and cytochrome P450 8B1(*cyp8b1*) was also observed in gibel carp receiving over 600 mg/kg dietary BA (*p* < 0.05; Figure 4). Interestingly, hepatic cytochrome P450 27A1 (*cyp27a1*) expression was lowest in the BA600 group and then increased to its peak in the BA800 group (*p* < 0.05; Figure 4).

### 3.4. Assembly of Transcriptome Sequencing and Identification of Differentially Expressed Genes (DEGs)

Based on the Illumina Hisseq-2500 platform, the hepatopancreatic samples from the BA0 and BA600 groups yielded an average of 51,395,362 raw reads, ranging from 41,170,334 to 62,836,908 per sample (Table 3 and Table S1). Clean reads of six samples ranged between 39,372,540 and 60,508,702 post-filtration, whose average Q20, Q30, and GC values were 97.28%, 91.14%, and 48.36%, respectively (Table 3 and Table S2). The average matching rate of clean reads with the known reference genome reached 43,034,580 (88.12%), being 87.95% (41,013,057) and 88.29% (45,056,104) for the BA0 group and BA600 groups, respectively (Table 3).

**Figure 5:**
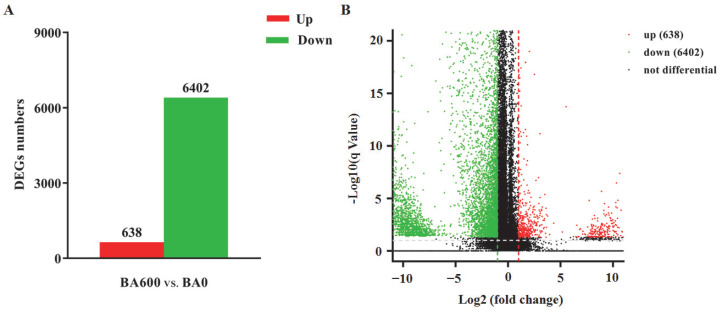
Identification of differentially expressed genes (DEGs) in the hepatopancreas of gibel carp between the BA600 and BA0 groups. Bar chart (**A**) and volcano plot (**B**) of DEGs in the comparison of BA600 vs. BA0. Genes with |log2(FC)| > 1 and corrected *p*-values < 0.05 are identified as DEGs and displayed in the diagram. The red bar/point, green bar/point, and black point represent up-regulated DEGs, down-regulated DEGs, and genes without expression difference, respectively.

Furthermore, 7040 DEGs were screened out, including 638 up-regulated and 6402 down-regulated DEGs (Figure 5, Table S3).

### 3.5. Functional Classification and Enrichment of DEGs Using GO and KEGG Analysis

**Figure 6:**
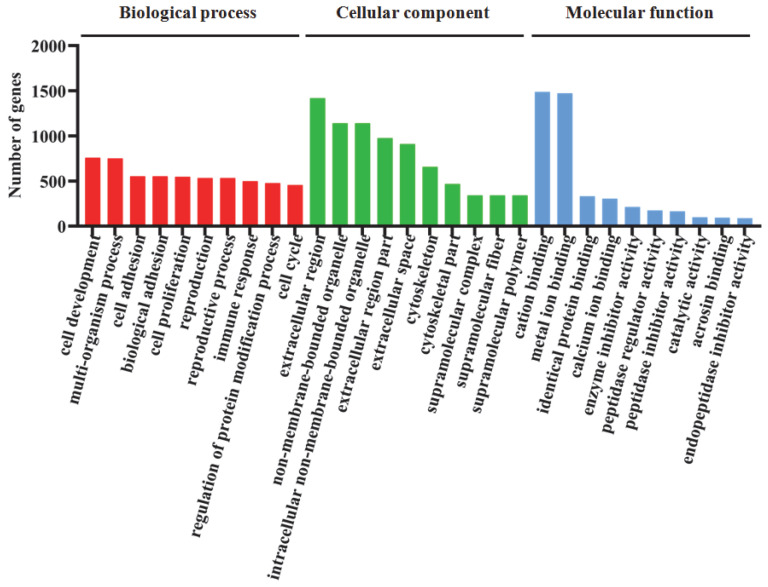
Gene ontology (GO) classification and enrichment of DEGs in the hepatopancreas of gibel carp between the BA600 and BA0 group. Significantly enriched GO terms of DEGs in the comparison of BA600 vs. BA0. *X*-axis represents the significantly enriched GO term. *Y*-axis represents the number of DEG significantly enriched in the GO term. The color and height of the bars represent three categories of GO terms and the number of DEGs significantly enriched in one GO term, respectively. GO terms with corrected *p*-values < 0.05 are screened, and only the top 10 significantly enriched GO terms are displayed.

Based on the GO database, the identified DEGs between BA0 and BA600 were divided into three ontologies, containing 208, 84, and 32 significantly enriched GO terms in the biological process (BP), cellular component (CC), and molecular function (MF), respectively (Figure 6, Table S4). Among the significantly enriched GO terms in BP, cell development (GO:0048468), multi-organism process (GO:0051704), cell adhesion (GO:0007155), and biological adhesion (GO:0022610) had the highest number of DEGs, containing 758, 749, 552, and 552 genes, respectively (Figure 6, Table S4). The enriched GO term with the highest DEG number in CC was extracellular region (GO:0005576) (Figure 6, Table S4). In MF, the top two enriched GO terms were cation binding (GO:0043169) and metal ion binding (GO:0046872) (Figure 6, Table S4).

**Figure 7:**
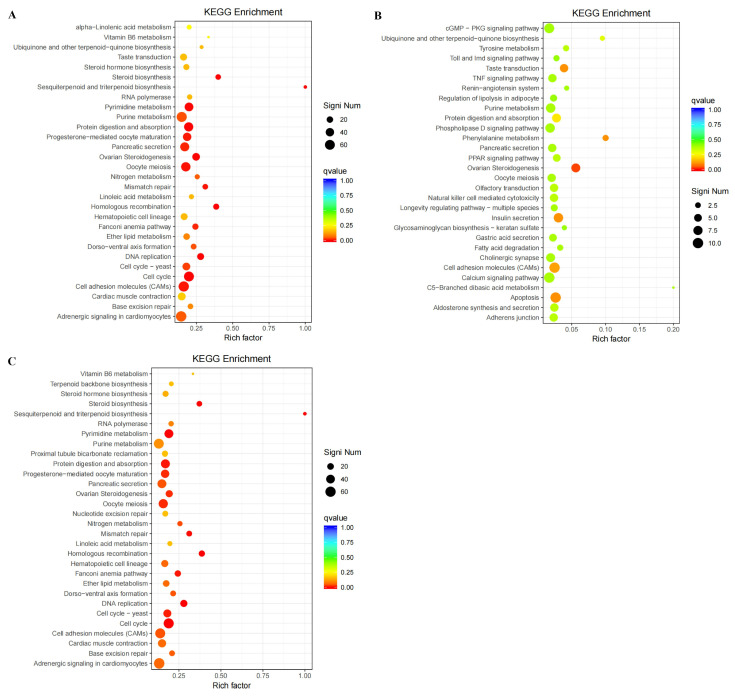
Kyoto Encyclopedia of Genes and Genomes (KEGG) enrichment analysis of DEGs in the hepatopancreas of gibel carp between the BA600 and BA0 groups. Significantly enriched KEGG pathways of DEGs (**A**), up-regulated DEGs (**B**), and down-regulated DEGs (**C**) in the comparison of BA600 vs. BA0. *X*-axis represents the proportion of DEGs annotated in a certain pathway relative to all DEGs. *Y*-axis represents the enriched KEGG pathway term. The color and area of the bubbles represent the enrichment degree and the number of DEGs enriched in one KEGG pathway, respectively. KEGG pathways with corrected *p*-values < 0.05 are screened, and the top 20 significantly enriched GO terms are displayed.

In the comparison of BA600 vs. BA0, there were 19 KEGG pathways with significant enrichment in the hepatopancreatic DEGs (Figure 7A, Table S5). These highly enriched pathways included cell cycle, ovarian steroidogenesis, steroid biosynthesis, protein digestion and absorption, etc. (Figure 7A, Table S5). Up-regulated DEGs were enriched in only one signaling pathway (Figure 7B, Table S5). However, the down-regulated DEGs were significantly linked to 18 known pathways, such as homologous recombination, cell cycle, steroid biosynthesis, protein digestion and absorption, and sesquiterpenoid and triterpenoid biosynthesis (Figure 7C, Table S5).

### 3.6. qRT-PCR Validation of DEGs

**Figure 8:**
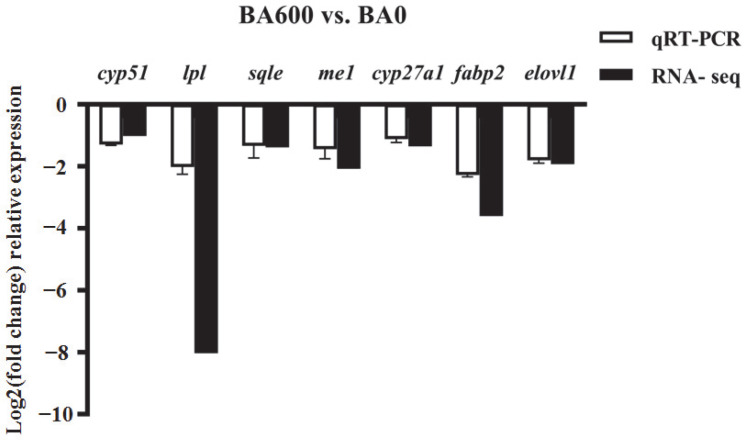
Verification of DEGs expression using qRT-PCR. Expression levels of 7 DEGs in the gibel carp hepatopancreas of BA600 vs. BA0 using qRT-PCR. The relative expression of each gene was measured by qRT-PCR and calculated for the expression of the candidate housekeeping genes (*ef1a* and *rpl13a*). All values are shown as mean ± SD (*n* = 3).

The expression patterns of seven randomly selected DEGs were highly consistent with the respective data of hepatopancreatic transcriptomes in gibel carp, exhibiting significant down-regulation (Figure 8).

## 4. Discussion

### 4.1. Effects of Dietary BA Supplementation on Serum Biochemical Parameters in Juvenile Gibel Carp Fed HFD

Serum biochemical indicators (such as AST and ALT) can reflect the health status and comprehensive function in the livers of fish and non-fish animals [4,27,28]. In this study, significantly lower serum ALT and, in particular, AST were observed in the BA400 and BA600 groups (*p* < 0.05), indicating an alleviation of HFD-induced liver injury compared to other BA concentrations. These data confirmed the previous findings that exogenous BA addition could reduce the enzyme activities of plasma ALT and AST in several fish species [16,17,29,30], especially when high-energy diets are used. However, when the BA supplemental level increased to 800 mg/kg in this study, serum AST increased to a level close to the control group. Significant increases in serum AST and ALT were also observed in striped catfish (*Pangasianodon hypophthalmus*) supplemented with 1500 mg/kg BA [29]. On the other hand, the addition of BA to the HFD had no significant effect on serum AST or ALT in grass carp [24]. Therefore, the mitigation effect of dietary BA on fish liver impairments caused by high-energy diets is closely related to its concentration and fish species.

With the increase in the dietary BA level, the HDL content first increased and then decreased, reaching the maximum value in the BA400 group (*p* < 0.05). LDL decreased and achieved its lowest value in the BA600 group (*p* < 0.05), which was inversely elevated above the control level in the BA800 group (*p* < 0.05). Similar results on serum HDL and LDL levels were demonstrated in Chinese perch (*Siniperca chuatsi*) [31] and black snapper (*Acanthopagrus schlegelii*) [32]. Considering the physiological roles of HDL and LDL in cholesterol homeostasis and their association with the risk of metabolic syndrome [33], relatively higher HDL and lower LDL levels in the serum of fish from the BA400 and BA600 groups indicated a significantly reduced cholesterol flux from the liver to the blood and extrahepatic tissues and a higher hepatic uptake/metabolism of dietary lipids, which could mitigate the possible metabolic risk in HFD-fed cyprinid fish [4]. This agrees with previous findings that exogenous BA and the relevant composition are more conducive to reducing hepatic lipid droplets in fish [16].

In line with the lipid-lowering effect of BAs in some freshwater and marine fish fed HFD [10,16,34,35], increasing dietary BA from 400 to 800 mg/kg in the HFD continuously decreased the serum TG and TC of gibel carp (*p* < 0.05). These lower contents in serum TG and TC herein suggested the declining incidence and severity of lipotoxicity and metabolic disorder caused by HFDs in fish [36,37]. Nevertheless, excessive doses of BA and its metabolites can unfavorably affect the physiological and metabolic responses involved in fish growth and health via the disruption of BA homeostasis within the enterohepatic circulation [16,38]. Elevated TC contents related to superabundant BA-induced hepatotoxicity were not significantly observed in this study, which could mainly be attributed to a broad range of BA tolerance in gibel carp, as also reported in omnivorous cyprinoid species [16,17].

### 4.2. Effects of Dietary BA Supplementation on Hepatopancreas Histology in Juvenile Gibel Carp Fed HFD

Consistent with the morphological changes in several HFD-fed fish treated with dietary BA [10,31,38,39], hepatic structure damages, including swollen cells, hazy cell margins, fat vacuolization, and inflammatory infiltration, could be relieved in gibel carp, especially those supplied with 400–600 mg/kg BA. But the liver exhibited cellular infiltration and more nuclear migration following higher levels of BA (800 mg/kg). This reversal morphology may partly be attributed to the upper tolerable limit of BA in gibel carp at less than 800 mg/kg. Similar findings on the recommended tolerance of BA, falling between 60 and 600 mg/kg, were reported in common carp [17]. Hepatocytes of Nile tilapia (*Oreochromis niloticus*) fed excessive doses of dietary BA (1350 mg/kg) also exhibited severe nuclear migration and vacuolation [40], which validated the above speculation to some extent. Moreover, the results of the serum biochemical parameters largely agreed with the liver histopathological observation.

### 4.3. Effects of Dietary BA Supplementation on Gene Expression Related to Lipid Metabolism in Juvenile Gibel Carp Fed HFD

The increasing inclusion of dietary BA caused the mRNA levels of *acc1*, *fas*, and *me1* genes to decrease to varying degrees. Fewer fatty acids and essential substrates derived from these lipogenic enzymes, particularly malonyl-CoA, palmitate, and nicotinamide adenine dinucleotide phosphate (NADPH), can be further converted into long-chain polyunsaturated fatty acids (LC-PUFAs) in gibel carp, which can be achieved by the elongase of very long-chain fatty acid (Elovl) family-mediated reactions [39,40]. Correspondingly, significantly lower *elovl4* occurred in the hepatopancreas, indicating a reduction in both the ability and yield of LC-PUFA synthesis in gibel carp fed HFDs with 0.02–0.06% BA. These findings agreed with the BA-induced attenuation of key genes in de novo lipogenesis in multiple aquatic animals fed HFDs [14,16,41,42]. But no change in hepatic *fas* was reported in HFD-fed grass carp regardless of BA supplementation [24]. It was mostly attributed to only one BA concentration utilized in HFD-fed grass carp, which might be inadequate to cause the elevation in *fas* expression.

In addition to PUFA biosynthesis, dramatically reduced *dgat*, which participates in the decisive step of triacylglycerol (TAG) biosynthesis through the Kennedy pathway or monoacylglycerol acyltransferase (MGAT) pathway [43], implied an attenuation of hepatic lipid accumulation in HFD-fed gibel carp, at least in the specific range of 400–600 mg/kg dietary BA. This speculation could be corroborated by lower hepatic TG content/lipid deposition in rodents and fish with functionally inhibited DGAT [43,44]. The down-regulation of multiple fatty acid synthesis-associated genes in our study and in other fish species [29,45,46] could also support this claim to some degree.

Herein, the *adipor 2* gene, a part of the adiponectin/AdipoR system with a function to modulate lipid homeostasis in fish via adiponectin receptors (Adipors) [47,48,49], was significantly up-regulated in gibel carp treated with dietary BA. Similarly, the obvious up-regulation of the hormone-sensitive lipase (*hsl*) gene occurred with the 0.06% to 0.08% supplementation of dietary BA. It affirmed the findings that BA supplementation accelerates lipid breakdown and fatty acid oxidation in fish liver [16,29], as reported in several fish species regardless of high or low dietary fat content. PPAR family member *α* and AMPK regulatory subunit β increased their transcript levels to varying degrees with increasing BA levels, respectively, whose expression broadly had a similar tendency to the up-regulation of the above-mentioned *adipor2* and *hsl*. Given that both PPARs and AMPK function as critical regulators of energy metabolism in fish and non-fish animals [4,50,51], it may be inferred that the addition of dietary BA might initiate fat decomposition and oxidation in the hepatopancreas of HFD-fed gibel carp by transcriptionally activating Adiponectin-PPARα, Adiponectin-AMPK, or other related pathways [48,49].

As demonstrated in other fish and non-fish animals [42,52], remarkably lower expressions of HMG-CoA reductase (Hmgcr) at the transcriptional level were found in fish receiving 400–800 mg/kg BA, causing reduced cholesterol production in the liver due to its function as a rate-limiting enzyme in cholesterol synthesis [42]. A block of endogenous BA biosynthesis would be expected, manifesting as the large down-regulation of cholesterol 7α-hydroxylase (*cyp7a1*) and sterol 12α-hydroxylase (*cyp8b1*) genes, which encode key enzymes in the synthesis of BA from cholesterol [16]. This repressed BA synthesis herein suggested less cholesterol conversion/catabolism within gibel carp hepatopancreases, which is mainly achieved by FXR-mediated classical signaling [53]. The lowest sterol-27-hydroxylase (*cyp27a1*) expression was found in gibel carp receiving 600 mg/kg BA, whereas this substantially increased to its maximum in the BA800 group, indicating the production of more chenodeoxycholic acid (CDCA) at higher doses of BA. The CDCA amount derived from the *cyp27a1*-triggered alternative pathway is very minimal, at only 10% of overall BA production [16,42], which might be inadequate to cover the overall decline in BA yields in HFD-fed fish treated with dietary BA. Thus, the increased availability of exogenous BA can enhance cholesterol clearance, transport, and lipid digestion within the liver [16], thereby lowering intrahepatic fat accumulation in the HFD-fed gibel carp. However, this favorable effect is likely reversed in the presence of excessive doses of dietary BA (higher than 600 mg/kg).

The dose-dependent BAs may execute lipid-lowering effects through multiple signals related to lipid metabolism [16], including AMPK/SREBP1 pathway, SREBP1/CYP7A1 pathway, and FXR/TGR5 axis. Moreover, the interactions of different organs and the cross-talk between BAs and microbiota could directly/indirectly regulate lipid metabolism-related pathways in the host by intestinal microbiota [16], which in turn modulates the actual effect of BA supplementation in fish. Thus, how these signaling pathways overlap/cross-talk with each other by specific genes requires more studies. Additionally, the potential risk caused by excessive BAs in gibel carp, such as the rebound in some BA synthesis genes (*cyp27a1*) in the BA800 group, and its molecular mechanism are still unclear and need further investigation.

### 4.4. Effects of Dietary BA Supplementation on Hepatopancreas Transcriptomic Profiles in Juvenile Gibel Carp Fed HFD

The dominantly enriched GO category of DEGs was biological process (BP), herein, similarly to the transcriptomic results in other fishes [54,55]. Furthermore, in the BP category, “cell development” was the top-enriched GO term with the greatest amount of DEGs, particularly up-regulated DEGs. Combined with the improved growth performance in the BA600 group (Table S7) and our previous report [22], these results may validate the growth-promoting effect of suitable exogenous BA in HFD-fed aquatic animals, which has been demonstrated in some cultured fish under HFD conditions [10,14,16,32].

In the KEGG enrichment analysis of all DEGs, 19 known pathways were identified and mainly involved in biosynthetic processes and metabolic responses, including “Steroid biosynthesis”, “Protein digestion and absorption”, and “Sesquiterpenoid and triterpenoid biosynthesis”. Moreover, down-regulated DEGs were highly enriched in 18 KEGG pathways, similarly to those of all DEGs. Only “Ovarian Steroidogenesis” was a significantly enriched pathway among the up-regulated DEGs. These results were partially substantiated by other research on dietary BA in aquatic animals [54,55,56], suggesting that the physiologic mechanism of BA in fish is achieved by modulating the nutrient metabolism and absorption, especially steroid-related metabolism.

“Steroid biosynthesis” and “Ovarian Steroidogenesis” were both notable categories associated with steroid metabolism-related processes, being among the top five significantly enriched pathways. Most DEGs in these two pathways were down-regulated, including *cyp51*, *cyp27a1*, and squalene monooxygenase (*sqle*) (Figure 7). Steroid biosynthesis signaling strongly participates in lipid homeostasis, in which many substrates and enzymes in the respective steps are lipid metabolism-related components [57,58]. Lanosterol 14α-demethylase (CYP51), a member of cytochrome P450 enzymes, is involved in sterol biosynthesis by providing substrates [57]. Herein, significantly lower *cyp51* expression implied the reduced catalytic reaction of lanosterol demethylation, causing less availability of the substrate for de novo cholesterol synthesis [57,59]. Actually, the exacerbated lanosterol accumulation induced by the depletion or suppression of *cyp51* mRNA/protein has been reported to diminish cholesterol content in vivo and in vitro [59,60]. Similarly, the down-regulated *sqle* gene, encoding the rate-limiting enzyme of cholesterol generation in the liver [58], suggested attenuated levels of hepatic cholesterol production and accumulation in BA-fed gibel carp. This speculation can be confirmed by the BA-induced decrease in serum TC and *hmgcr* mRNA detected above. Furthermore, DEGs involved in cholesterol transport, such as *lpl*, were obviously decreased in the hepatopancreas of BA-fed gibel carp. Considering that cholesterol is a key sterol type of lipid component and a precursor for conversion to steroids and BAs in animals [37,58], our findings indicated that cholesterol synthesis is endogenously impeded by the addition of dietary BA. This could be supported by several previous works, in which steroid metabolism-related genes were repressed in the liver of fish under BA and its relevant component-supplementing conditions [10,58].

## 5. Conclusions

BA supplementation at 400–600 mg/kg could effectively reverse multiple serum biochemical parameters linked with liver metabolism and damage in gibel carp under HFD conditions, along with the alleviation of liver histological anomalies. Most genes involved in lipogenesis and cholesterol synthesis were dramatically down-regulated in HFD-fed gibel carp with the BA addition of 400–600 mg/kg (particularly 600 mg/kg BA), whereas gene expressions of lipid decomposition and transport were increased. Based on the Illumina platform, 7040 DEGs were selected in the hepatopancreas of BA600 vs. BA0 and were functionally enriched in the pathways mainly associated with metabolic and biosynthetic responses, particularly steroid metabolism signaling. Overall, dietary BA with an optimal concentration (400–600 mg/kg) can both diminish fat and the relevant metabolite synthesis and accelerate lipolysis and fatty acid transport in the hepatopancreas of gibel carp, thereby minimizing HFD-triggered harmful effects in fish.

## Figures and Tables

**Table 1:** Formulation and proximate composition of experimental diets (%dry matter).

Ingredient	Group				
	BA0	BA200	BA400	BA600	BA800
Defatted fish meal	15.00	15.00	15.00	15.00	15.00
Soybean meal	23.00	23.00	23.00	23.00	23.00
Cottonseed meal	18.00	18.00	18.00	18.00	18.00
Canola meal	11.80	11.80	11.80	11.80	11.80
Flour	17.00	16.98	16.96	16.94	16.92
Ca(H_2_PO_4_)_2_	1.80	1.80	1.80	1.80	1.80
Lys	0.15	0.15	0.15	0.15	0.15
Met	0.05	0.05	0.05	0.05	0.05
Choline	0.30	0.30	0.30	0.30	0.30
Bile acid	0.00	0.02	0.04	0.06	0.08
Soybean oil	12.00	12.00	12.00	12.00	12.00
Vitamin premix	0.40	0.40	0.40	0.40	0.40
Mineral premix	0.50	0.50	0.50	0.50	0.50
Total	100.00	100.00	100.00	100.00	100.00
Nutrient levels (%)					
Crude protein	37.57 ± 0.17	37.34 ± 0.18	37.80 ± 0.22	37.75 ± 0.01	37.98 ± 0.49
Crude fat	12.50 ± 0.12	12.37 ± 0.21	12.20 ± 0.20	12.29 ± 0.18	12.37 ± 0.22
Crude ash	7.99 ± 0.60	7.97 ± 0.70	8.00 ± 0.73	8.07 ± 0.14	8.15 ± 0.93
Wet	10.48 ± 0.24	10.28 ± 0.06	10.06 ± 0.20	10.31 ± 0.23	10.70 ± 0.16

Note: Vitamin and mineral premix (each/kg diet): VA, 5000 IU; VD, 2000 IU; VE, 50 mg; VB1, 8 mg; UK, 5 mg; VB2, 10 mg; VB12, 0.03 mg; VB6, 8 mg; inositol, 100 mg; folic acid, 3 mg; pantothenic acid, 30 mg; nicotinic acid, 30 mg; biotin, 0.4 mg; VC, 180 mg; Cu, 4 mg; Fe, 170 mg; Zn, 150 mg; Mn, 22 mg; I, 1 mg; Co, 0.25 mg; Se, 0.4 mg; Mg, 300 mg.

**Table 2:** Primer information for qRT-PCR.

Gene	Forward Primer (5′–3′)	Reverse Primer (5′–3′)	Size (bp)
*ef1a*	CGCCAGTGTTGCCTTCGT	CGCTCAATCTTCCATCCCTT	98
*rpl13a*	CTTCTGGAGGACAGTAAGAGGTATGC	GGAGGAGGGATGCCATCAAAGAC	96
*acc1*	AAATGTTTCGCAATGAACGAG	ATCTTGATATACTCTGCGTTGG	85
*adipor2*	CGAAAAGGAGGAGAAAACA	CTTCAGCCAATCAGGGAG	172
*ampkβ1*	TGGAGCTCGACCCAAAATCC	AACACAGTGGGCCTTTCCTC	141
*cyp7a1*	ACCTCGGTTGTGCTCTTCAG	GGACATACTGCCCAGCAATC	192
*cyp8b1*	CGACCGTTTTCTCACACC	GTTCCTGCTCCCCAAG	96
*cyp27a1*	CCCACTGGTGATCTGGTCTC	GTCCAGTTTGGCAGGAACAC	170
*cyp51*	CGGAGAAACACGACGACA	GCCAGGAAGAAGCCCA	160
*dgat*	CTCAGTTAGCCGTGTTCTTC	TCTGTGCCATCATTCCC	105
*elovl1*	CCTTCTTCTTCGTGCTGT	CTGTTGGGTGTTCTTTGG	80
*elovl4*	GGCTTCTAATCTACTCTCCT	GTTCATTGGTTCCTTGTG	104
*fabp2*	CCTTTGACTATTCTCTGGC	TTTCCGTTGTCCTTGC	100
*fas*	GGCCAAGAGAATCTACTGCAC	TGATGGGAATGTCACCCCTT	95
*hmgcr*	ATTCCCAGAGCCCACG	TGCTTTCCATCCAATAACAG	144
*hsl*	GAAGAGTGTTTCTATGCCTACT	CCGTGAGACATTGCCCTCAT	140
*lpl*	AGTACGCAGATGCCCAAAG	CTGGCCTCTGAATCCCAATAC	111
*me1*	TTGTGCTCTTCCACTTCTG	TGGCTGGGTTTCCGAC	224
*pparα*	AAGAACCGAAACAAATGCCAA	AACCTCAGCTTCTCCGACT	110
*sqle*	CAAACTCTTGACTACATTCCC	CCCTCTTTCGCTTTACATC	104

Note: The primers used in this study were designed using Primer Premier 6 software. *ef1a*: elongation factor 1 alpha; *rpl13a*: ribosomal protein L13a; *acc1*: acetyl CoA carboxylase 1; *adipor2*: adiponectin receptor 2; *ampkβ1*: AMP-activated protein kinase β1; *cyp7a1*: cytochrome P450 7A1, also named as cholesterol 7α-hydroxylase; *cyp8b1*: cytochrome P450 8B1, also named as sterol 12α-hydroxylase; *cyp27a1*: cytochrome P450 27A1, also named as sterol-27-hydroxylase; *cyp51*: cytochrome P450 51, also named as lanosterol 14α-demethylase; *dgat*: diacylglycerol acyltransferase; *elovl1*: elongase of very long-chain fatty acids 1; *elovl4*: elongase of very long-chain fatty acids 4; *fabp2*: fatty acid binding protein 2; *fas*: fatty acid synthase; *hmgcr*: 3-hydroxy-3-methylglutaryl-CoA reductase; *hsl*: hormone-sensitive lipase; *lpl*: lipoprotein lipase; *me1*: malic enzyme 1; *pparα*: peroxisome proliferator-activated receptor α; *sqle*: squalene monooxygenase.

**Table 3:** Summary of transcriptome sequencing and assembly in the hepatopancreas of gibel carp.

Items	BA0-1	BA0-2	BA0-3	BA600-1	BA600-2	BA600-3
Raw reads	47,142,592	49,939,382	51,110,188	56,172,768	41,170,334	62,836,908
Clean reads	45,190,484	48,071,420	49,105,916	54,077,826	39,506,686	60,591,842
Clean bases	6,165,204,891	6,325,647,847	6,778,769,014	7,329,021,946	5,427,859,827	8,268,273,878
Q20 (%)	97.00	97.47	97.16	97.24	97.13	97.39
Q30 (%)	90.45	91.83	90.89	91.20	90.83	91.65
GC (%)	48.63	48.15	48.01	48.71	48.04	48.62
Total mapping ratio (%)	87.94	87.88	88.02	88.32	87.98	88.56
Uniquely mapping ratio (%)	79.44	79.32	80.30	80.01	80.15	80.38

Note: BA0 (1–3) represents three samples from the BA0 group; BA600 (1–3) represents three samples from the BA600 group.

## Data Availability

All data regarding the findings in this study are available from the corresponding author upon reasonable request.

## Supplementary Materials

The following supporting information can be downloaded at https://www.mdpi.com/article/10.3390/ani15192853/s1, Table S1: Statistics of raw transcriptome data in the hepatopancreas of gibel carp from the comparison of BA600 vs. BA0; Table S2: Statistics of clean transcriptome data in the hepatopancreas of gibel carp from the comparison of BA600 vs. BA0; Table S3: Statistics of DEGs in the hepatopancreas of gibel carp from the comparison of BA600 vs. BA0; Table S4: GO analysis of DEGs in the hepatopancreas of gibel carp from the comparison of BA600 vs. BA0; Table S5: KEGG enrichment analysis of DEGs in the hepatopancreas of gibel carp from the comparison of BA600 vs. BA0; Table S6: Information on seven lipid metabolism-related DEGs analyzed by RNA-seq; Table S7: Growth parameters of gibel carp fed with HFDs containing 0 mg/kg and 600 mg/kg BA for 8 weeks. Reference [22] is cited in the Supplementary Materials.
