# Supplementary material for: Dietary Bile Acid Influences the Physiological, Morphological, Lipid Metabolism-Related Responses, and Transcriptomic Profile of Hepatopancreas in High-Fat Diet-Fed Juvenile Gibel Carp (Carassius auratus gibelio)
Source: Animals (Basel). 2025 Sep 30;15(19):2853. doi: 10.3390/ani15192853 (PMC12523780; doi:10.3390/ani15192853)
Supplement: Supplementary file 1 [file animals-15-02853-s001.zip › Table S1, S2, S3, S6, S7.pdf]

Table S1 Statistics of raw transcriptome data in the hepatopancreas of gibel carp from the comparison of BA600 vs. BA0.

Sample	Raw reads	Total bases (bp)	Average read length (bp)	Q20(%)	Q30(%)	GC(%)
BA0_1	47142592	7071388800	150	94.87	86.93	48.99
BA0_2	49939382	7666528200	150	95.49	88.46	48.63
BA0_3	51110188	7490907300	150	95.17	87.60	48.32
BA600_1	56172768	8425915200	150	95.20	87.84	49.08
BA600_2	41170334	6175550100	150	95.02	87.36	48.38
BA600_3	62836908	9425536200	150	95.51	88.52	48.96

Note:

Hepatopancreatic samples in the control group were named as BA0, feeding on soybean oil (SO)-based high-fat diet (HFD) supplemented with 0 mg/kg bile acid (BA). Hepatopancreatic samples in the experimental groups were named as BA600, feeding on SO-based HFD supplemented with 600 mg/kg BA. Three replicates were used for each group in the transcriptome analysis.

Table S2 Statistics of clean transcriptome data in the hepatopancreas of gibel carp from from the comparison of BA600 vs. BA0.

Sample	Clean reads	Clean bases (bp)	Average read length (bp)	Q20(%)	Q30(%)	GC(%)	Total reads	Total mapped	Mutiple mapped	Uniquely mapped
BA0_1	43554360	6165204891	136.43	97.00	90.45	48.63	43554360	38303528(87.94%)	3702223(8.50%)	34601305(79.44%)
BA0_2	47424808	6325647847	131.59	97.47	91.83	48.15	47424808	41675061(87.88%)	4059403(8.56%)	37615658(79.32%)
BA0_3	48923400	6778769014	138.04	97.16	90.89	48.01	48923400	43060581(88.02%)	3773758(7.71%)	39286823(80.30%)
BA600_1	53149330	7329021946	135.53	97.24	91.20	48.71	53149330	46943044(88.32%)	4415674(8.31%)	42527370(80.01%)
BA600_2	39372540	5427859827	136.46	97.13	90.83	48.04	39372540	34638533(87.98%)	3080583(7.82%)	31557950(80.15%)
BA600_3	60508702	8268273878	137.39	97.39	91.65	48.62	60508702	53586735(88.56%)	4947098(8.18%)	48639637(80.38%)

Note:

Hepatopancreatic samples in the control group were named as BA0, feeding on soybean oil (SO)-based high-fat diet (HFD) supplemented with 0 mg/kg bile acid (BA). Hepatopancreatic samples in the experimental groups were named as BA600, feeding on SO-based HFD supplemented with 600 mg/kg BA. Three replicates were used for each group in the transcriptome analysis.

Table S3 Statistics of DEGs in the hepatopancreas of gibel carp from the comparison of BA600 vs BA0.

Group	DEGs		
	Up-regulated	Down-regulated	Total
BA600 vs BA0	638	6402	7040

Note:

Hepatopancreatic samples in the control group were named as BA0, feeding on soybean oil (SO)-based high-fat diet (HFD) supplemented with 0 mg/kg bile acid (BA). Hepatopancreatic samples in the experimental groups were named as BA600, feeding on SO-based HFD supplemented with 600 mg/kg BA. DEGs between two groups were screened at |log2(fold change)| >1 and corrected $P < 0.05$.

Table S6 Information on seven lipid metabolism-related DEGs analyzed by RNA-seq.

Gene ID	Name	MeanTPM (BA600)	MeanTPM (BA0)	log2FoldChange //qValue	Gene Description
LOC113046101	*cyp27a1*	9.9510	25.4394	-1.35↓ // 0	5-beta-cholestane-3-alpha
LOC113062450	*cyp51*	26.5386	53.9632	-1.02↓ // 0	lanosterol 14-alpha demethylase
LOC113045626	*elovl1*	0.1578	0.8056	-2.35↓ // 0	elongation of very long chain fatty acids protein 1-like
LOC113084692	*fabp2*	0.1333	1.6267	-3.61↓ // 0	fatty acid-binding protein 2, intestinal-like
LOC113064829	*lpl*	0.0001	0.0933	-9.87↓ // 0	lipoprotein lipase-like
LOC113053102	*me1*	0.4600	1.9433	-2.08↓ // 0	NADP-dependent malic enzyme-like
LOC113059233	*sqle*	6.2148	16.2571	-1.39↓ // 0	squalene monooxygenase-like

Note:

Hepatopancreatic samples in the control group were named as BA0, feeding on soybean oil (SO)-based high-fat diet (HFD) supplemented with 0 mg/kg bile acid (BA). Hepatopancreatic samples in the experimental groups were named as BA600, feeding on SO-based HFD supplemented with 600 mg/kg BA. Gene names (|log2(fold change)| >1 and corrected $P < 0.05$) and fold changes in the transcript abundances of these genes were analyzed by RNA-seq.

Abbreviations for Table S6 were as follows:

cyp27a1: cytochrome P450 27A1; *cyp51*: cytochrome P450 51; *elovl1*: elongase of very long-chain fatty acids 1; *fabp2*: fatty acid binding protein 2; *lpl*: lipoprotein lipase; *me1*: malic enzyme 1; *sqle*: squalene monooxygenase.

Table S7 Growth parameters of gibel carp fed with HFDs containing 0 mg/kg and 600 mg/kg BA for 8 weeks.

Group	Initial weight (g)	Final weigh (g)	Weight gain rate (%)	Survival rate (%)	Feed conversion ratio
BA0	32.38±0.16	71.93±4.44	149.31±14.42	100	1.76±0.11
BA600	32.65±0.18	86.09±2.46*	180.59±4.46*	100	1.31±0.06*

Note:

All value are shown as mean ± SD (n=3) and further evaluated via t-test. Column with an asterisk (*) represents a significant difference between the BA0 and BA600 group ($P < 0.05$).

Data in Table S7 have been published by our research team in a Chinese journal (DOI: 10.13557/j.cnki.issn1002-2813.2023.14.009).

Reference List:

Zhang, J.; Chen, X.; Qu, L.; Xia, T.; Ding, H.; Xu, Y.; Xu, J.; Cheng, H. Effect of Bile Acids in High-Lipid Diet on Growth Performance and Muscle Fatty Acid Composition of Gibel Carp (*Carassius auratus* Gibelio). *Feed Res.* **2023**, *46*, 41–45. https://doi.org/10.13557/j.cnki.issn1002-2813.2023.14.009.
